# Efficacy and Recovery of Remimazolam Versus Midazolam in Sedated Colonoscopy: A Multicenter Randomized Controlled Trial in Japan

**DOI:** 10.1111/den.70130

**Published:** 2026-02-27

**Authors:** Daisuke Yamaguchi, Ryoji Ichijima, Hisatomo Ikehara, Yosuke Minoda, Mitsuru Esaki, Ayako Takamori, Akiyoshi Yoh, Moeko Shirouzu, Kento Sadashima, Yutaro Fujimura, Takuya Shimamura, Hironobu Takedomi, Takashi Akutagawa, Nanae Tsuruoka, Yasuhisa Sakata, Takuya Wada, Chika Kusano, Ryo Shimoda, Motohiro Esaki

**Affiliations:** ^1^ Division of Gastroenterology, Department of Internal Medicine, Faculty of Medicine Saga University Saga Japan; ^2^ Department of Gastroenterology Saiseikai Kawaguchi General Hospital Kawaguchi Japan; ^3^ Endoscopy Division National Cancer Center Hospital Tokyo Japan; ^4^ Department of Gastroenterology, Internal Medicine Kitasato University School of Medicine Sagamihara Japan; ^5^ Department of Medicine and Bioregulatory Science, Graduate School of Medical Sciences Kyushu University Fukuoka Japan; ^6^ Clinical Research Center Saga University Hospital Saga Japan; ^7^ Clinical Research Center in Hiroshima Hiroshima University Hospital Hiroshima Japan; ^8^ Department of Endoscopic Diagnostics and Therapeutics Saga University Hospital Saga Japan

**Keywords:** colonoscopy, midazolam, Modified Observer's Assessment of Alertness/Sedation scale, remimazolam, sedation

## Abstract

**Objectives:**

Sedation during colonoscopy is becoming increasingly important. Remimazolam, an ultra‐short‐acting benzodiazepine, has a shorter pharmacokinetic half‐life than that of midazolam. This study examined whether remimazolam provides superior sedation during colonoscopy in Japanese patients.

**Methods:**

This prospective, multicenter, randomized, single‐blind, controlled trial included adults (18–80 years) scheduled for sedated colonoscopy. Participants were randomized to the remimazolam and midazolam groups. The primary outcome was the proportion of ambulatory patients 5 min after colonoscopy. Secondary outcomes were successful pre‐procedure sedation (Modified Observer's Assessment of Alertness/Sedation [MOAA/S] ≤ 4), recovery time, total sedative dose, and adverse events.

**Results:**

Forty patients were enrolled and analyzed (remimazolam, *n* = 19; midazolam, *n* = 21). At 5 min post‐colonoscopy, ambulation was achieved in 100% (19/19) of remimazolam patients and 19.1% (4/21) of midazolam patients (*p* < 0.0001). The median time [interquartile range (IQR)] from procedure end to full alertness (MOAA/S = 5) was 0 [0–0] min for remimazolam and 10 [5–20] min for midazolam (*p* < 0.0001). The median time [IQR] from procedure end to independent ambulation was 0 [0–5] min for remimazolam and 20 [10–30] min for midazolam (*p* < 0.001). Pre‐procedure sedation was successful (MOAA/S ≤ 4) in 100% of both groups. The median amount [IQR] of total sedative dose was 5 [4–6] mg for remimazolam and 3 [3] mg for midazolam. Hypoxemia occurred in 5.3% and 9.5% of patients in the remimazolam and midazolam groups, respectively.

**Conclusions:**

Compared with midazolam, remimazolam resulted in significantly faster recovery after colonoscopy in Japanese patients, with comparable achievement of target sedation and a low incidence of hypoxemia.

**Clinical Registration:**

Trial number: jRCTs071240062

**Abbreviations**
ASA‐PS: American Society of Anesthesiologists physical status
MOAA/S: Modified Observer assessment of alertness/sedation

## 1 Introduction

Colonoscopy is a crucial procedure for the early detection of colorectal cancer, and the demand for sedation during colonoscopies continues to increase, making it an integral part of routine clinical practice [1]. The “Guidelines for Sedation in Gastrointestinal Endoscopy (2nd Edition),” issued by the Japanese Society of Gastroenterological Endoscopy [2] emphasize that sedation improves patient acceptance and satisfaction in both upper gastrointestinal endoscopy and colonoscopy, thereby supporting better diagnostic and therapeutic outcomes. Consequently, the proportion of sedated endoscopies performed in Japan has been increasing [2, 3, 4, 5, 6].

However, compared to many Western countries, Japan has fewer sedatives covered by the national health insurance, leading to continued reliance on the off‐label use of benzodiazepines. Midazolam is the drug most commonly used in routine clinical practice [2, 7, 8, 9, 10]. However, its relatively long half‐life can cause residual effects after the procedure, potentially delaying discharge from the endoscopy suite and prolonging stay in the recovery room [11, 12]. Given the limited number of recovery beds available after endoscopy, such delays can limit broader use of sedation. Remimazolam, an ultra‐short‐acting benzodiazepine approved by the US Food and Drug Administration for procedural sedation, received Japanese approval on June 24, 2025, for sedation during gastrointestinal endoscopy and was subsequently listed for national health insurance reimbursement on August 14, 2025. Since remimazolam has a shorter pharmacokinetic half‐life (t½) than midazolam (0.75 h vs. 4.29 h), remimazolam is anticipated to reduce post‐endoscopy recovery and time to discharge [13, 14, 15, 16, 17, 18].

We previously reported the efficacy and safety of remimazolam in randomized, placebo‐controlled trials involving Japanese patients [19, 20], and multiple international studies have demonstrated its safety and effectiveness in endoscopy [21, 22, 23, 24, 25, 26]. Furthermore, retrospective propensity score‐matched studies comparing remimazolam with midazolam and reports of remimazolam efficacy in small Japanese endoscopy cohorts have been published [17, 27, 28, 29]. However, to the best of our knowledge, no prospective randomized controlled trial comparing remimazolam and midazolam for colonoscopy in Japanese patients has been published.

This manuscript reports the colonoscopy cohort of a prospective, multicenter, randomized controlled trial registered as jRCTs071240062. Under the same registered protocol, the upper gastrointestinal endoscopy cohort has been reported separately [30]. The cohorts were mutually exclusive and analyzed independently by procedure type, and procedure‐specific analyses were prespecified in the study protocol.

## 2 Methods

### 2.1 Study Design

We conducted a prospective, multicenter, randomized, single‐blind, active‐controlled study at four Japanese institutions: Saga University Hospital, Saiseikai Kawaguchi General Hospital, Kitasato University Hospital, and Kyushu University Hospital. Adults scheduled for outpatient colonoscopy under sedation were enrolled after providing written informed consent. Treatment allocation was centralized via the UMIN INDICE cloud platform using a minimization algorithm with stratification by site, age (18–69 vs. 70–80 years), and sex. Participants were blinded to the assigned sedative, while investigators were responsible for safety oversight, constituting a single‐blind design. Screening was performed within 28 days before the procedure and follow‐up was performed the day after colonoscopy. This study was prospectively registered with the Japan Registry of Clinical Trials (jRCT; jRCTs071240062; registered on September 26, 2024; https://jrct.niph.go.jp/). All procedures adhered to the Declaration of Helsinki and the CONSORT guidelines. The upper gastrointestinal endoscopy cohort from the same registered protocol has been reported separately [30].

### 2.2 Eligibility Criteria

The inclusion criteria were as follows: (1) written informed consent provided, (2) Japanese adults aged 18–80 years; and (3) patients scheduled to undergo colonoscopy under sedation.

Exclusion criteria were as follows: (1) planned therapeutic endoscopy (e.g., polypectomy, endoscopic mucosal resection, or endoscopic submucosal dissection); (2) history of upper or lower GI surgery; (3) receipt of dialysis; (4) daily alcohol consumption ≥ 60 g; (5) presence of severe hepatic dysfunction; (6) American Society of Anesthesiologists physical status (ASA‐PS) III–V; (7) inability to ambulate independently; (8) regular use of benzodiazepines, analgesics, or other central nervous system depressants; (9) use of any sedative within the previous 4 weeks; (10) diagnosis of acute angle‐closure glaucoma; (11) diagnosis of myasthenia gravis; (12) pregnancy or breastfeeding; (13) hypersensitivity to benzodiazepines or flumazenil or other contraindications; and (14) any condition judged by the investigator to warrant exclusion.

### 2.3 Study Procedures and Sedative Dosing

The participants were randomized in a 1:1 ratio to receive remimazolam or midazolam. Remimazolam (50‐mg vial reconstituted with 50 mL saline to 1 mg/mL) was administered as an initial 3‐mg IV bolus, with 1‐mg supplemental boluses as needed. These doses were based on previous studies [16, 19, 20]. Midazolam (10 mg/2 mL diluted with 8 mL saline to 1 mg/mL) was administered as an initial 2‐mg IV bolus, followed by 1‐mg supplemental boluses as required. The initial dosage of midazolam was determined based on the public disclosure dosage (0.03 mg/kg) of off‐label use for gastrointestinal endoscopy in Japan. All boluses were delivered over at least 15 s and separated at intervals of at least 2 min, with a maximum cumulative dose of 10 mg (combined preprocedural and intraprocedural).

### 2.4 Sedation Assessment and Intraprocedural Management

Sedation depth was assessed using the Modified Observer's Assessment of Alertness/Sedation (MOAA/S) scale [31]. MOAA/S was recorded ≥ 2 min after each bolus. Colonoscopy began once adequate sedation was achieved (MOAA/S ≤ 4). If, after reaching the maximum total dose, the MOAA/S score remained 5, the sedative was deemed ineffective and the procedure continued without further study medication (“sedation failure”). During colonoscopy, signs of arousal—MOAA/S of 5 or clinically meaningful movement—triggered maintenance boluses per the same algorithm, without exceeding 10 mg in total.

### 2.5 Recovery, Discharge, and Follow‐Up

After scope withdrawal, the MOAA/S scores were recorded at 0, 5, 10, 20, and 30 min and then every 10 min until the score reached 5. At that point, a standardized gait test was performed in which patients were asked to walk 5 m in a straight line without staggering, and the time from the end of the procedure to ambulation was recorded. Patients remained in the recovery room for at least 30 min and left the colonoscopy suite only after achieving MOAA/S = 5 and satisfactory ambulation. For safety, all patients were accompanied home by family or friends and instructed not to drive a car on the day of the procedure or on the following day. Satisfaction and interval events were evaluated by telephone the following day.

### 2.6 Study Endpoints

The primary endpoint was the proportion of participants who were able to ambulate independently 5 min after completion of colonoscopy (“effective sedation”); this definition was aligned with the provisions for successful sedation used in a prior investigator‐initiated phase III trial in Japan [19].

The secondary endpoints comprised successful preprocedure sedation defined as MOAA/S ≤ 4; the dose required to achieve pre‐procedure sedation; the total dose required to complete colonoscopy; the time from the first dose to the start of the procedure (i.e., attainment of MOAA/S ≤ 4); the time from the end of the procedure to the decision to begin walking (MOAA/S = 5); the total time from procedure end to completion of walking (including rest periods); patient satisfaction at discharge and at next‐day telephone follow‐up by the investigators at each facility, each assessed on a 5‐point scale; and endoscopist satisfaction on a 5‐point scale.

### 2.7 Definitions

Two physicians were deployed in the endoscopy room: the endoscopist who performed the colonoscopy assessed the MOAA/S score during the procedure. After the colonoscopy, the endoscopist who performed the procedure assessed walking ability. The other physician assessed and intervened when unexpected adverse events occurred. In the recovery room, the MOAA/S score was assessed by nurses. All procedures were performed by certified specialists from the Japan Gastroenterological Endoscopy Society. All the participating physicians held the current American Heart Association Basic Life Support certification. Colonoscopy requires a thorough examination to reach the cecum. Loss of consciousness was defined as MOAA/S ≤ 1 at two consecutive assessments. Supplemental oxygen (2–5 L/min) was provided if SpO_2_ fell below 94%. The airway was secured when the respiratory rate dropped below 5 breaths/min; if spontaneous breathing did not recover, bag‐valve‐mask ventilation was initiated. Respiratory rate was monitored using electrocardiogram‐derived impedance or visual observation, similar to other vital signs. Flumazenil was administered for an urgent reversal. Adverse event severity was graded according to the Common Terminology Criteria for Adverse Events, version 5.0. After colonoscopy, patients and endoscopists were asked to grade overall satisfaction (grade 1–5; best scored as 5) encompassing sedation effects, recovery effects, and adverse events.

### 2.8 Sample Size

Based on prior reports [16, 19, 20] and assuming ambulation at 5 min in 80% of remimazolam recipients versus 35% of midazolam recipients (two‐sided α = 0.05; power = 80%), a total of 36 participants (18 per group) were required. Allowing for approximately 10% attrition and balanced enrollment across the four sites, the planned sample size was 40 participants (20 per group). Interim analyses were not performed.

### 2.9 Statistical Analysis

Continuous variables were presented as mean ± standard deviation (SD) and compared between groups using the Student's *t*‐test. When a non‐normal distribution was suspected, median (IQR; interquartile range) was shown and the Mann–Whitney *U* test was applied. Categorical variables are summarized as counts (percentages) and compared using the chi‐square test or Fisher's exact test. For endpoints, either proportions or between‐group differences were estimated, along with their 95% confidence intervals (CIs). Questionnaire data are reported as a distribution of 5‐point ratings. Statistical significance was set at *p* < 0.05. Analyses were performed using JMP Student Edition version 18.0.0 (SAS Institute, Cary, NC, USA).

## 3 Results

### 3.1 Baseline Characteristics

**FIGURE 1:**
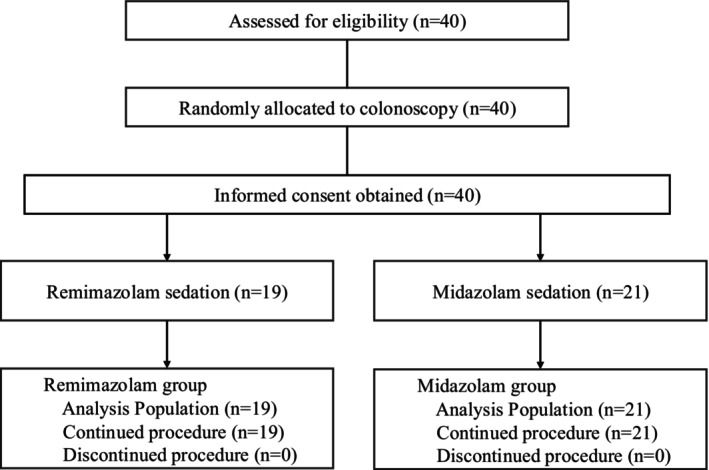
Flow diagram of the study.

Figure 1 depicts patient flow. Forty patients were analyzed (remimazolam, *n* = 19; midazolam, *n* = 21). Table 1 summarizes the baseline characteristics and comorbidities of patients. The two groups were generally balanced, with no significant differences in age (55.0 [43.0–74.0] vs. 60.0 [52.0–68.0] years, *p* = 0.431), sex (male 52.6% vs. 52.4%, *p* = 0.987), body mass index (23.0 ± 3.7 vs. 23.1 ± 4.0 kg/m^2^, *p* = 0.912), smoking history (15.8% vs. 28.6%, *p* = 0.334), or major comorbidities. The ASA‐PS class differed, with a higher proportion of class I patients in the remimazolam group than in the midazolam group (I/II: 57.9%/42.1% vs. 19.1%/80.9%, *p* = 0.011).

**TABLE 1:** Baseline characteristics.

	Remimazolam group	Midazolam group	*p*
Number of patients (*N*)	19	21	
Age (years)	55.0 [43.0, 74.0]	60.0 [52.0, 68.0]	0.431
Sex, males	10 (52.6%)	11 (52.4%)	0.987
Height (cm)	165.0 ± 9.7	163.6 ± 9.3	0.636
Weight (kg)	62.6 ± 11.5	62.2 ± 14.3	0.924
BMI (kg/m^2^)	23.0 ± 3.7	23.1 ± 4.0	0.912
ASA‐PS, I	11 (57.9%)	4 (19.1%)	0.011
ASA‐PS, II	8 (42.1%)	17 (80.9%)	
Smoking history	3 (15.8%)	6 (28.6%)	0.334
Comorbidities			
Hypertension	4 (21.1%)	9 (42.9%)	0.142
Dyslipidemia	5 (26.3%)	6 (28.6%)	1.000
Diabetes mellitus	4 (21.1%)	3 (14.3%)	0.574
Mild lung disease	0 (0.0%)	1 (4.8%)	1.000
GI malignancy	0 (0.0%)	3 (14.3%)	0.233

*Note:* Results are presented as the number of patients or mean ± standard deviation. When a non‐normal distribution was suspected, median (IQR; interquartile range) and *p* value by the Mann–Whitney *U* test is shown.

Abbreviations: ASA‐PS, American Society of Anesthesiologists physical status; BMI, body mass index; GI, gastrointestinal.

### 3.2 Sedation Rate (Primary Endpoint)

Five minutes after completion of colonoscopy, independent ambulation was achieved by 100.0% (19/19) in the remimazolam group versus 19.1% (4/21) in the midazolam group (χ^2^ = 26.7, *p* < 0.0001). The 95% confidence intervals were 82.4%–100.0% for remimazolam and 0.1%–41.9% for midazolam (Table 2).

**TABLE 2:** Percentage of patients who achieved ambulation 5 min after endoscopy.

Groups	Ambulatory: not ambulatory	Percentage of patients who responded to sedation	95% CI	χ^2^	*p*
Remimazolam	19:0	100.0%	82.4%–100.0%	26.7	< 0.0001
Midazolam	4:17	19.1%	0.1%–41.9%		

*Note:* This difference was statistically significant (χ^2^ = 26.7, *p* < 0.0001).

### 3.3 Assessment of the Efficacy of Sedation

Preprocedure sedation (MOAA/S ≤ 4) was attained in all patients in both groups. As shown in Table 3, the time from first dose to procedure start did not differ significantly (2 [2–6] vs. 2 [2–4] min, *p* = 0.722), and procedure duration was comparable (12 [10–16] vs. 12 [10–12] min, *p* = 0.814). Recovery was substantially faster with remimazolam: the time from procedure end to MOAA/S = 5 was 0 [0–0] min versus 10 [5–20] min (*p* < 0.0001), and the time to independent ambulation was 0 [0–5] min vs. 20 [10–30] min, respectively (*p* < 0.001).

**TABLE 3:** Times to achieve sedation and to achieve ambulation after endoscopy.

	Group	*n*	Mean	SD	Median [IQR]	Mean difference	[95% CI]	*p*
Time to start endoscopy start (min)	Remimazolam	19	3.89	2.62	2 [2, 6]	0.56	[−0.84, 1.97]	0.722
	Midazolam	21	3.33	1.71	2 [2, 4]			
Procedure time (min)	Remimazolam	19	12.42	4.68	12 [10, 16]	0.61	[−2.09, 3.31]	0.814
	Midazolam	21	11.81	3.74	12 [10, 12]			
Time from endoscopy end to MOAA/S score = 5 (min)	Remimazolam	19	0.53	1.58	0 [0, 0]	−17.09	[−26.44, −7.75]	< 0.0001
	Midazolam	21	17.62	20.04	10 [5, 20]			
Total time from ending endoscopy to achieving ambulation (min)	Remimazolam	19	1.58	2.39	0 [0, 5]	−22.71	[−31.13, −14.28]	< 0.0001
	Midazolam	21	24.29	17.98	20 [10, 30]			

*Note:* *p* values are based on the Mann–Whitney *U* tests.

### 3.4 Sedative Dosing

Table 4 details sedative requirements. Doses to achieve/maintain sedation differed between groups: pre‐colonoscopy 3 [3–5] mg versus 2 [2, 3] mg (*p* = 0.0004), intraprocedural 1 [0–2] mg versus 0 [0–1] mg (*p* = 0.007), and total 5 [4–6] mg versus 3 [3] mg (*p* < 0.0001) for remimazolam and midazolam, respectively.

**TABLE 4:** Sedative dose required for colonoscopy.

	Group	*n*	Mean	SD	Median [IQR]	Mean difference	[95% CI]	*p*
Pre‐colonoscopy dosage	Remimazolam	19	3.95	1.31	3 [3, 5]	1.28	[0.58, 1.98]	0.0004
	Midazolam	21	2.67	0.86	2 [2, 3]			
During colonoscopy dosage	Remimazolam	19	1.21	1.03	1 [0, 2]	0.78	[0.21, 1.35]	0.007
	Midazolam	21	0.43	0.75	0 [0, 1]			
Total dosage	Remimazolam	19	5.16	1.80	5 [4, 6]	2.06	[1.14, 2.98]	< 0.0001
	Midazolam	21	3.10	1.00	3 [3, 3]			

*Note:* *p* values are based on the Mann–Whitney *U* tests.

### 3.5 Safety Assessments

The adverse events are summarized in Table 5. Supplemental oxygen was required for 1/19 (5.3%) and 2/21 (9.5%) patients who received remimazolam and midazolam, respectively. Hypertension occurred in 1/19 (5.3%) and 1/21 (4.8%) patients, respectively, and no hypotension or headache was observed. None of the patients required flumazenil or bag‐valve‐mask ventilation during colonoscopy, and no grade III–V adverse events were reported.

**TABLE 5:** Adverse events.

	Remimazolam group (*n* = 19)	Midazolam group (*n* = 21)
Adverse event (Grades I/II/III–V)		
Oxygen required	1 (5.3%) (0/1/0)	2 (9.5%) (2/0/0)
Hypertension	1 (5.3%) (1/0/0)	1 (4.8%) (0/1/0)
Hypotension	0 (0.0%) (0/0/0)	0 (0.0%) (0/0/0)
Flumazenil administration	0 (0.0%) (0/0/0)	0 (0.0%) (0/0/0)
Manual ventilation	0 (0.0%) (0/0/0)	0 (0.0%) (0/0/0)
Headache	0 (0.0%) (0/0/0)	0 (0.0%) (0/0/0)

### 3.6 Patients' and Endoscopists' Assessments

As shown in Table 6, patient satisfaction was high in both groups (5 [4, 5] vs. 5 [4, 5]; *p* = 0.961), with no significant difference. Endoscopist satisfaction was likewise high (5 [5] vs. 5 [3–5]; *p* = 0.192), with no significant between‐group difference.

**TABLE 6:** Patients' and endoscopists' assessments.

	Group	*n*	Mean	SD	Median [IQR]	Mean difference	[95% CI]	*p*
Patients' assessment	Remimazolam	19	4.47	1.02	5 [4, 5]	−0.05	[−0.64, 0.54]	0.961
	Midazolam	21	4.52	0.81	5 [4, 5]			
Endoscopists' assessment	Remimazolam	19	4.63	0.96	5 [5, 5]	0.54	[−0.22, 1.29]	0.192
	Midazolam	21	4.10	1.34	5 [3, 5]			

*Note:* As a non‐normal distribution was suspected, median [IQR] is also shown, and *p* value is derived from the Mann–Whitney *U* test.

## 4 Discussion

To the best of our knowledge, this trial is the first randomized, multicenter, head‐to‐head comparison of remimazolam and midazolam during colonoscopy in Japan. Prior domestic comparisons were limited to nonrandomized retrospective analyses and propensity score‐matched studies [26, 27], underscoring the added value of this trial's design and endpoint selection. With remimazolam approved in Japan in June 2025 for gastrointestinal endoscopic sedation, a direct comparison with the established midazolam standard at this time is of considerable clinical significance.

We previously demonstrated the safety of remimazolam in Phase III trials [19]. In the present study, we aimed to assess the recovery efficacy of remimazolam compared to midazolam, which is considered a major advantage of this sedative. We thus set the primary endpoint as “ambulation after 5 min” to evaluate recovery efficacy following colonoscopy, as in the previous study [19]. This endpoint is important because quick arousal after sedative colonoscopy can improve safe and smooth management of the recovery room. The primary endpoint (ability to walk unaided within 5 min after the procedure) was achieved in 100% of the patients in the remimazolam group and 19.1% in the midazolam group. All patients in the remimazolam group were able to walk within 5 min after colonoscopy, which is consistent with a previous report [19]. Although a greater proportion of patients in the remimazolam group were ASA‐PS class I, the markedly higher rate of early ambulation compared with that in the midazolam group indicated that remimazolam clearly accelerated recovery of consciousness. Clinically, remimazolam facilitates post‐colonoscopy recovery and shortens the time to ambulation, allowing patients to walk immediately after the procedure.

Initial sedation success before colonoscopy (MOAA/S ≤ 4) was 100% in both groups, aligning with the high success rates reported in international trials comparing remimazolam and midazolam [13, 14, 15]. In this study, recovery metrics favored remimazolam; while time to achieve preprocedure sedation and procedure duration did not differ between groups, both the interval from scope withdrawal to MOAA/S = 5 (0.53 min vs. 17.62 min with remimazolam and midazolam, respectively) and the time from procedure completion to ambulation (1.58 vs. 24.29 min with remimazolam and midazolam, respectively) were significantly shorter with remimazolam. As the demand for colonoscopy sedation to reduce patient discomfort and improve endoscopist satisfaction increases, the associated need for recovery spaces and additional monitoring personnel also increases [2, 32]. The use of remimazolam may help mitigate these operational pressures, and its adoption in sedated colonoscopy, including in outpatient settings, is expected to increase.

In this trial, the protocol‐specified initial doses differed between groups (remimazolam 3 mg and midazolam 2 mg), which explains the higher preprocedural dose in the remimazolam arm. During colonoscopy, additional 1‐mg boluses were administered using the same titration algorithm in both groups (≥ 2‐min intervals; maximum cumulative dose 10 mg). Despite the same titration protocol, participants in the remimazolam group more frequently required intraprocedural supplementation, which may reflect remimazolam's shorter duration of effect and the need for closer titration to maintain moderate sedation. When doses were additionally assessed on a molar basis (Table S1), the between‐group difference was small, suggesting broadly similar clinical sedative potency under the present titration strategy. However, the shorter elimination half‐life of remimazolam forced more active intraprocedural titration, potentially increasing the administration complexity; this trade‐off between rapid recovery after colonoscopy and practical burden for maintaining sedation should be clinically assessed in the future.

The safety outcomes were generally favorable. The remimazolam group required oxygen supplementation less frequently, and none of the patients in either group required flumazenil or bag‐valve‐mask ventilation. The low rate of supplemental oxygen use suggests a favorable respiratory profile in routine practice, potentially reducing the need for staff intervention. Moreover, the availability of flumazenil as an antagonist of remimazolam and midazolam provides reassurance to endoscopists. Patient and endoscopist satisfaction were high in both groups, suggesting that sedation‐assisted colonoscopy may reduce anxiety and improve adherence to endoscopic evaluations.

Overall, these results suggest that recovery room occupancy time after colonoscopy can be reduced, potentially decreasing the staffing burden and improving colonoscopy suite throughput. Accordingly, remimazolam appears promising, but larger confirmatory trials are needed.

This study has some limitations. First, the modest sample size may not have captured rare adverse events. Second, although patients were blinded, endoscopists and nurses were not; we judged blinding endoscopists and nurses was impractical considering the need for safe drug administration and appropriate intraprocedural management, whereas this study design might have caused bias, such as additional drug administration. Third, the significantly higher proportion of ASA‐PS II patients in the midazolam group may have influenced the results. Fourth, because this trial focused on colonoscopy, the findings may not be generalizable to longer therapeutic procedures or to higher‐risk populations (e.g., ASA‐PS ≥ III, older adults). Finally, remimazolam, a newly approved agent, is currently more expensive than midazolam in the Japanese reimbursement setting; the average cost per patient was JPY 397.3 for remimazolam and JPY 17.8 for midazolam in this study. Separately, from a “green endoscopy” perspective, medication waste and its environmental impact (e.g., leftover volumes influenced by vial size/packaging and preparation practices) warrant evaluation in real‐world sedation workflows.

In summary, a randomized, multicenter trial in Japan demonstrated that remimazolam yielded significantly shorter recovery times than midazolam, while maintaining equivalent rates of initial sedation success and potentially reducing the need for supplemental oxygen. These findings suggest that remimazolam is an effective benzodiazepine with operational advantages for colonoscopic sedation in Japan.

## Author Contributions

D.Y. conceived and designed the study and drafted the manuscript. M.E. was a major contributor to the manuscript writing. A.T. contributed to improving the methodology from a statistical perspective. R.I., H.I., Y.M., M.E., A.Y., M.S., K.S., Y.F., T.S., H.T., T.A., N.T., Y.S., T.W., C.K., and R.S. designed the study and revised the manuscript. All the authors have read and approved the final version of this manuscript.

## Funding

The corresponding author has received research funding from Mundipharma K.K. (Tokyo, Japan).

## Ethics Statement

This study was approved by the Certified Review Board of Saga University Hospital (approval number C20240401), and the implementation plan was submitted to the Ministry of Health, Labor, and Welfare of Japan. The principal investigator must obtain approval from the administrator of the medical institution to conduct a clinical study. Written informed consent was obtained from all the study participants (jRCT; no. jRCTs071240062).

## Consent

The authors have nothing to report.

## Conflicts of Interest

Mundipharma funded this study; however, the sponsor had no role in study design, data collection, analysis, or interpretation. The original developer of remimazolam is PAION AG, while Mundipharma is the licensee and distributor in Japan.

## Supporting information


**Table S1:** Molecular weight of sedative dosage required for colonoscopy.

## Data Availability

The datasets used and/or analyzed in the current study are available from the corresponding author upon reasonable request.
