# Supplementary material for: Efficacy and Recovery of Remimazolam Versus Midazolam in Sedated Colonoscopy: A Multicenter Randomized Controlled Trial in Japan
Source: Dig Endosc. 2026 Feb 27;38(3):e70130. doi: 10.1111/den.70130 (PMC12946854; doi:10.1111/den.70130)
Supplement: Supplementary file 1 — Table S1: Molecular weight of sedative dosage required for colonoscopy. [file DEN-38-0-s001.docx]

**Supplementary Table 1.** Molecular weight of sedative dosage required for colonoscopy

|  | Group | n | Mean | SD | Median [IQR] | Mean difference | [ 95% CI] | p-value |
| --- | --- | --- | --- | --- | --- | --- | --- | --- |
| Pre-endoscopy dosage | Remimazolam | 19 | 8.99 | 2.99 | 6.83 [6.83, 11.39] | 0.79 | [-1.01, 2.59] | 0.166 |
| (μmol) | Midazolam | 21 | 8.21 | 2.63 | 6.15 [6.15, 9.23] |  |  |  |
| During endoscopy dosage | Remimazolam | 19 | 2.76 | 2.35 | 2.28 [0, 4.56] | 1.44 | [-0.05, 2.93] | 0.053 |
| (μmol) | Midazolam | 21 | 1.32 | 2.30 | 0 [0, 3.08] |  |  |  |
| Total dosage | Remimazolam | 19 | 11.75 | 4.11 | 11.39 [9.11, 13.67] | 2.23 | [-0.08, 4.53] | 0.215 |
| (μmol) | Midazolam | 21 | 9.52 | 3.06 | 9.23 [9.23, 9.23] |  |  |  |

Remimazolam = 439 g/mol, Midazolam = 325 g/mol

P values are based on the Mann–Whitney U tests.
